# Polymorphisms in regulatory regions of Cyclooxygenase-2 gene and breast cancer risk in Brazilians: a case-control study

**DOI:** 10.1186/1471-2407-10-613

**Published:** 2010-11-08

**Authors:** Diogo N Piranda, Juliana S Festa-Vasconcellos, Laura M Amaral, Anke Bergmann, Rosane Vianna-Jorge

**Affiliations:** 1 Divisão de Farmacologia, Coordenação de Pesquisa Instituto Nacional do Câncer - INCA, RJ, Brazil; 2 Instituto de Ciências Biomédicas, Universidade Federal do Rio de Janeiro - UFRJ, RJ, Brazil; 3 Serviço de Fisioterapia, Hospital do Câncer III - INCA, RJ, Brazil

## Abstract

**Background:**

Cyclooxygenase-2 (COX-2) is up-regulated in several types of cancer, and it is hypothesized that COX-2 expression may be genetically influenced. Here, we evaluate the association between single-nucleotide polymorphisms (SNPs) in the COX-2 gene (*PTGS2*) and the occurrence of breast cancer among Brazilian women.

**Methods:**

The study was conducted prospectively in two steps: First, we screened the promoter region and three fragments of the 3'-untranslated region of *PTGS2 *from 67 healthy Brazilians to identify SNPs and to select those with a minor allele frequency (MAF) of at least 0.10. The MAF of these selected SNPs was further characterized in 402 healthy volunteers to evaluate potential differences related to heterogeneous racial admixture and to estimate the existence of linkage disequilibrium among the SNPs. The second step was a case-control study with 318 patients and 273 controls designed to evaluate *PTGS2 *genotype- or haplotype-associated risk of breast cancer.

**Results:**

The screening analysis indicated nine SNPs with the following MAFs: rs689465 (0.22), rs689466 (0.15), rs20415 (0.007), rs20417 (0.32), rs20419 (0.015), rs5270 (0.02), rs20424 (0.007), rs5275 (0.22) and rs4648298 (0.01). The SNPs rs689465, rs689466, rs20417 and rs5275 were further studied: Their genotypic distributions followed Hardy-Weinberg equilibrium and the MAFs were not affected by gender or skin color. Strong linkage disequilibrium was detected for rs689465, rs20417 and rs5275 in the three possible pairwise combinations. In the case-control study, there was a significant increase of rs5275TC heterozygotes in cases compared to controls (OR = 1.44, 95% CI = 1.01-2.06; P = 0.043), and the haplotype formed by rs689465G, rs689466A, rs20417G and rs5275C was only detected in cases. The apparent association with breast cancer was not confirmed for rs5275CC homozygotes or for the most frequent rs5275C-containing haplotypes.

**Conclusions:**

Our results indicate no strong association between the four most frequent *PTGS2 *SNPs and the risk of breast cancer.

## Background

Cyclooxygenases (COXs) are key enzymes in mediating the conversion of free arachidonic acid into prostaglandin H_2_, the precursor of molecules such as prostaglandins, prostacyclin and thromboxanes [1]. Two isoforms of cyclooxygenase (COX-1 and COX-2) are known. The constitutive cyclooxygenase (COX-1) is present in many tissues and synthesizes prostaglandins involved in maintaining normal tissue homeostasis [2]. The inflammatory enzyme COX-2 is not detected in most normal tissues but can be induced by cytokines, growth factors or tumor promoters. COX-2 catalyzes the synthesis of prostaglandins, such as prostaglandin E_2 _(PGE_2_), which can affect cell proliferation, apoptosis and angiogenesis [3], contributing to tumor progression. COX-2 is present in several types of solid tumors and, in breast cancer, is associated with parameters of aggressiveness, including tumor size, positive nodal status and lower survival [4,5]. In addition, inhibition of COX-2 by nonsteroidal anti-inflammatory drugs has been associated with a protective effect against a variety of cancers [6] and may be effective in the prevention and treatment of breast cancer [7,8].

The mechanisms involved in the regulation of COX-2 expression remain unclear and may be influenced by genetic variations. The human COX-2 gene, *PTGS2*, is located on chromosome 1 (locus q25.2-q25.3), is 8.3 kb in size, contains 10 exons and produces an mRNA of 4.6 kb. The analysis of the promoter region (PR) reveals the existence of several potential regulatory elements, including a TATA box and transcription binding sites for NF-kB, NF-IL6, AP-1, AP-2, GAS, TBP and cAMP response element. Several genetic variants have been described in regions next to these regulatory sites that may affect enzyme expression [9,10] and contribute to a greater risk of developing cancer.

In addition to variations in the PR, sites in the 3'-untranslated region (3'-UTR) of the gene may also be associated with increased risk of developing cancer. The 3'-UTR of the *PTGS2 *gene contains 30 AUUUA elements. Such repetitions generate consensus binding sequences for proteins and inflammatory mediators that regulate the stability and degradation of mRNA [11-13]. These repeats are also present in other genes encoding inflammatory mediators (cytokines and proto-oncogenes) whose mRNAs are very unstable [14]. Genetic variations in the 3'-UTR of the *PTGS2 *gene may contribute to increased stability of mRNA and the synthesis of COX-2.

The frequency of SNPs in the *PTGS2 *gene may vary between different ethnic groups [15,16]. No data are available on the frequency of such variant forms in the Brazilian population, either in healthy subjects or in cancer patients. The high rate of racial admixture, with a major contribution from Europeans and Africans in the formation of the Brazilian population, suggests that the variant forms of the *PTGS2 *gene may have a high prevalence in Brazil and that their occurrence may lead to haplotypes with different potentials for changes in COX-2 expression.

In the present study, we identified single-nucleotide polymorphisms (SNPs) in the PR and 3'-UTR of the *PTGS2 *gene and evaluated their association with breast cancer occurrence among Brazilians.

## Methods

### Experimental Design and Study Population

This study was conducted prospectively in two steps: first, we screened 1.5 kb of the PR and three fragments comprising 1.2 kb of the 3'-UTR of the *PTGS2 *gene in 67 healthy Brazilians to identify *PTGS2 *SNPs and to select those with a minor allele frequency (MAF) of at least 0.10. The frequency of these selected SNPs was further characterized in 355 other healthy volunteers (comprising a total of 402) to evaluate potential differences in allelic distribution due to heterogeneous racial admixture. We adopted the classification scheme used in the 2000 Brazilian Census [17], which relies on self-perception of skin color. Accordingly, the individuals were distributed into the following three color groups: white, black and intermediate. The term "color" (*cor *in Portuguese) is preferred to "race" in Brazil because it captures the continuous aspects of phenotypes and also because a racial descent rule is not operational in this country [18]. The color stratification was not intended as an accurate ethnic classification. Instead, our objective was to evaluate potential differences in the frequency distribution of *PTGS2 *SNPs to ascertain if an independent population control would be necessary in the case-control study.

The second step was a case-control study, designed to evaluate the genotype-associated risk of breast cancer for the most prevalent *PTGS2 *SNPs, i.e., those with at least 0.10 MAF. This case-control study involved 318 women with breast cancer and 273 healthy controls. The patients had a confirmed diagnosis of breast cancer based on histopathological evaluation and were under current treatment at the Brazilian National Cancer Institute. The patients were assigned a recruitment interview when scheduled for routine blood exams. The controls were non-related healthy women with no signs or symptoms of breast cancer who were recruited among patients' escorts, hospital staff and blood donors of the Brazilian National Cancer Institute. The recruitment of both patients and controls occurred between January and October 2008.

All volunteers were informed about the procedures of the study and gave written consent to participate. Patients and controls were interviewed by trained personnel using a questionnaire to determine demographic and lifestyle characteristics. Information on clinical history was obtained from medical records for patients (N = 250) and collected in an additional questionnaire for controls (N = 183). The study was approved by the Ethics Committee of the Brazilian National Cancer Institute (Protocol #116/07).

### SNP Screening and Genotyping

Peripheral blood samples (3 mL) were collected from all subjects (volunteers, cases and controls). DNA was extracted using the DNAzol system (Invitrogen Life Technologies, Carlsbad, USA), following the procedures recommended by the manufacturer and were used to search for SNPs of the *PTGS2 *gene (GenBank accession #AY382629). The blood samples were kept at 4°C until DNA extraction, which was performed within 24 h of blood collection.

The genotyping analyses were performed by denaturing high-performance liquid chromatography (dHPLC), using the Wave™ DNA Fragment Analysis System (Transgenomic, Omaha, NE) or by PCR-RFLP (all enzymes from New England Biolabs). Table 1 summarizes the PCR conditions, the sets of primers and the enzymes used for each analysis.

**Table 1:** Nucleotide PRIMER sequences and PCR conditions for genotyping by dHPLC or PCR-RFLP

PRIMER Name	Nucleotide sequence				Region	Identified SNP	Number of cycles	Melting Temperature	**Enzyme**^**#**^
**-1290AG**	F	5'	TGCTGTCATTTTCCTGTAATGC	3'	PR	rs689465	34	60°C	*Pci*I
**-1195AG**	R	5'	TTTCTCTCCCTGATGCGTGG	3'		rs689466			*Alu*I

AM1*	F	5'	GCTGTCAAAATCTCCCTTCC	3'	PR	rs20415	30	58°C	
	R	5'	CCACGCATCAGGGAGAGAAA	3'					

AM2*	F	5'	AACCAAAATAATCCACGC	3'	PR		30	63°C	
	R	5'	CAAGGAGGGGGTGAAG	3'					

**-765GC**	F	5'	CTTTGTCCATCAGAAGGCAGG	3'	PR	rs20417	35	63°C	*Aci*I
	R	5'	TAGAGGGTCGAGGAAGTCACG	3'					

AM3*	F	5'	TTACCTTTCCCGCCTCTC	3'	PR	rs20419	30	58°C	
	R	5'	GCGTCGTCACTAAAACATAAAAC	3'					

AM4*	F	5'	GCTATGTATGTATGTGCTGC	3'	PR		31	55°C	
	R	5'	GAACTGGCTCTCGGAAGC	3'					

AM5*	F	5'	CGGTATCCCATCCAAGGC	3'	PR	rs5270	31	55°C	
	R	5'	CAGTGAGCGTCAGGAGCA	3'		rs20424			

**8473TC**	F	5'	CTGTTGCGGAGAAAGGAGTC	3'	3'-UTR	rs5275	30	58°C	
	R	5'	TCAAACAAGCTTTTACAGGTGA	3'					

9850AG**	F	5'	CGTTCCCATTCTAATTAATGCCCTT	3'	3'-UTR	rs4648298	34	50°C	*Alu*I
	R	5'	TGTGTCAAGCACTGTGGGTTTTAAT	3'					

**10335GA**	F	5'	TTTGGGAAGAGGGAGAAAATGA	3'	3'-UTR	rs689469	30	56°C	
	R	5'	TATGCGAATGTTTCAGTGCC	3'					

* 15; ** 16; PR: Promoter Region; 3'-UTR: 3';Untranslated Region

^# ^Enzymes used for PCR-RFLP. All other analyses were performed by dHPLC.

In the case of dHPLC analysis, all samples with chromatographic profiles suggestive of variation in the gene sequence were analyzed using ABI PRISM-377 equipment (TaqMan, PE Biosystems, Foster City, CA, USA). A portion of controls (10% of the samples) was also analyzed by automatic sequencing, and the results matched completely.

The four SNPs selected for the case-control study were genotyped with the same sets of primers used in the screening step. The SNPs rs689465, rs689466 and rs20417 could not be identified by dHPLC and were genotyped by PCR-RFLP (Table 1).

### Statistical Analysis

Allelic and genotypic frequencies were derived by gene counting and the adherence to the Hardy-Weinberg principle was evaluated by the chi-square test for goodness-of-fit. The evaluation of pairwise linkage disequilibrium was performed using the Fisher exact test, available on-line in GENEPOP (http://genepop.curtin.edu.au/; [19]), whereas the haplotype patterns were inferred using Haploview (http://www.broadinstitute.org/haploview; [20]), based on the algorithm of expectation and maximization. Comparisons of demographic and clinical features and of genotypic and haplotypic distributions between patients and controls were performed using the chi-square test for proportions. Univariate logistic regression analyses were performed to identify independent factors influencing the risk of developing breast cancer, which was estimated by the odds ratio (OR) with 95% confidence interval (95% CI). The threshold for significance was set at P < 0.05 (Pearson P-value). The clinically relevant factors with independent effects on breast cancer risk (OR and 95% CI >1) were used to create a multivariate final model using the Enter method. All statistical analyses were conducted using SPSS 13.0 for Windows (SPSS Inc., Chicago, Illinois).

## Results

The screening analysis of the PR and 3'-UTR of the *PTGS2 *gene in 67 healthy Brazilians revealed the existence of nine SNPs, which occurred with the following MAFs: *-1290AG *(rs689465, 0.22); *-1195AG *(rs689466, 0.15); -*1131GA *(rs20415, 0.007); *-765GC *(rs20417, 0.32), -*604TC *(rs20419, 0.015), -*163CG *(rs5270, 0.02), -*62CG *(rs20424, 0.007), *8473TC *(rs5275; 0.22) and *9850AG *(rs4648298; 0.01). The SNP *10335GA *(rs689469), which was reported to have a MAF of 0.02 among the Spanish [16], was not found.

The SNPs rs689465, rs689466, rs20417 and rs5275, which showed a higher than 0.10 MAF in this first population subset, were selected for further characterization in a larger sample (Table 2). No differences were observed in the allelic frequencies due to gender or skin color for any of the SNPs studied. All genotypic distributions followed Hardy-Weinberg equilibrium. Our results indicate strong pairwise linkage disequilibrium involving SNPs rs689465, rs20417 and rs5275 in the three possible combinations (Table 3). In fact, the minor alleles of these three SNPs often occurred simultaneously, whereas the rs689466 *G *allele occurred mostly as an isolated variation.

**Table 2:** Minor allelic frequency (MAF) of *PTGS*2 SNPs in Brazilians

SNP	Population	Alleles (N)	MAF (95%CI)	P χ2
**rs689465 AG**			**rs689465 G**	
	Total *	710	0.17 (0.14-0.20)	
				
	Men	222	0.20 (0.15-0.25)	0.273
	Women	488	0.16 (0.13-0.19)	
				
	Whites	298	0.18 (0.14-0.22)	0.87
	Intermediates	248	0.16 (0.11-0.20)	
	Blacks	164	0.16 (0.11-0.22)	
				
**rs689466 AG**			**rs689466 G**	
	Total *	710	0.13 (0.10-0.15)	
				
	Men	220	0.16 (0.12-0.21)	0.221
	Women	490	0.12 (0.09-0.15)	
				
	Whites	298	0.12 (0.08-0.16)	0.728
	Intermediates	246	0.13 (0.09-0.17)	
	Blacks	166	0.15 (0.10-0.21)	
				
**rs20417 GC**			**rs20417 C**	
	Total *	772	0.30 (0.27-0.33)	
				
	Men	242	0.28 (0.26-0.38)	0.783
	Women	528	0.29 (0.25-0.33)	
				
	Whites	328	0.30 (0.25-0.35)	0.949
	Intermediates	276	0.29 (0.23-0.34)	
	Blacks	168	0.31 (0.24-0.38))	
				
**rs5275 TC**			**rs5275 C**	
	Total *	698	0.30 (0.26-0.33)	
				
	Men	208	0.29 (0.23-0.35)	0.882
	Women	490	0.30 (0.26-0.34)	
				
	Whites	296	0.27 (0.22-0.32)	0.53
	Intermediates	250	0.31 (0.25-0.36)	
	Blacks	154	0.34 (0.26-0.41)	

* Differences in values are due to missing data (no PCR amplification).

P χ2: Chi-square test (Pearson P-value); 95%CI: 95% Confidence Interval

**Table 3:** Pairwise linkage disequilibrium between PTGS2 SNPs in Brazilians

SNP	χ2	P (Fisher)	[D']*	R2**
rs689465 & rs689466	3.159	0.206	99	4
rs689465 & rs20417	infinity	<0.0001	77	31
rs689466 & rs20417	6.860	0.032	62	3
rs689465 & rs5275	infinity	<0.0001	51	13
rs689466 & rs5275	1.095	0.579	49	2
rs20417 & rs5275	infinity	<0.0001	44	18

* D' (degree of imbalance) in module; ** R2 (degree of correlation).

The next step was a case-control study, conducted to examine the association between SNPs rs689465, rs689466, rs20417 and rs5275 and the occurrence of breast cancer. Cases and controls were first compared with regard to the distribution profile of clinical aspects potentially implicated in the risk of developing cancer and that could interfere as confounding factors on the analysis of the risk associated to *PTGS2 *SNPs (Table 4). A significant difference between cases and controls was found only for age (OR = 1.72, 95% CI = 1.24-2.39; P = 0.001).

**Table 4:** Impact of clinical and demographic characteristics on the risk of breast cancer in Brazilian women

Characteristic	Category	Cases	Controls	OR	95%CI	P χ2
Age* (years)	< 48	147	163	1		
	≥ 48	171	110	1.72	(1.24-2.39)	**0.001**
Missing data		0	0			
**Menarche (Age)**	< 12	120	93	1		
	≥ 12	120	74	1.25	(0.84-1.86)	0.258
Missing data		78	106			
Menopause (Age)**	≤ 54	98	62	1		
	≥ 55	10	5	0.79	(0.25-2.42)	0.68
Missing data		127	103			
**First Birth (Age)**	≤ 30	161	103	1		
	≥ 31 or nulliparous	75	68	1.41	(0.93-2.13)	0.09
Missing data		82	102			
**Fertile Time¥**	< 35	45	35	1		
	≥ 35	60	27	1.73	(0.83-3.42)	0.089
Missing data		3	5			
**Use of contraceptives **†	< 2	110	105	1		
	≥2	92	59	1.49	(0.97-2.27)	0.065
Missing data		116	109			
**Use of HRT**^**θ**^	no	79	43	1		
	yes	25	20	0.68	(0.32-1.44)	0.276
Missing data		4	4			
**Use of NSAIDs**	no	187	154	1		
	yes	31	13	1.96	(0.99-3.88)	0.053
Missing data		100	106			
**BMI**	Underweight: ≤ 18.4	9	1	4.13	(0.50-33.6)	0.28
	Normal: 18.5 - 24.9	98	45	1		
	Overweight: ≥ 25.0	159	63	1.15	(0.73-1.83)	0.55
Missing Data		52	164			
**Smoking Habit**	no	174	83	1		
	yes	98	38	1.23	(0.77-1.94)	0.42
Missing data		46	152			
**Color**	White	131	120			0.619‡
	Intermediate	125	96			
	Black	62	57			
Missing data		0	0			

* 48 years old is the median of cases + controls; ** Menopausal status: postmenopausal: cases n = 108, controls: n = 67; premenopausal: cases: n = 83, controls n = 103; ¥ Age of Menopause - Age of Menarche (only for women in menopause); † For at least 2 years; ^θ^HRT: Hormonal Reposition Therapy (only for women in menopause); NSAIDs: Non-Steroidal Anti-Inflammatory Drugs; BMI: Body Mass Index at diagnosis (patients) or at recruitment (controls), BMI = weight (Kg)/height^2^ (m^2^); OR: Odds Ratio; 95%CI: 95% Confidence Interval; P from Chi-square test (Pearson p-value); ‡ P from Fisher test.

The genotypic distributions of *PTGS2 *SNPs in cases and controls are shown in Table 5. Our results indicate a significant difference in the distribution of rs5275 genotypes, with a higher frequency of heterozygotes among cases than among controls (OR = 1.44, 95% CI = 1.01-2.06; P = 0.043). To control possible confounding variables that may have influenced the observed association measures, a multivariate regression model was built. The best model, combining the effects of age and the rs5275 SNP, showed a higher risk for rs5275 heterozygotes (OR_adjusted _= 1.43, 95% CI = 1.00-2.06; P = 0.049). The analysis of rs5275 SNP under recessive or dominant models did not confirm the risk association for breast cancer (data not shown).

**Table 5:** Genotypic distribution of *PTGS2 *SNPs in cases and controls

SNP	Population	N*	Genotypic Distribution N (Freq)			P χ2
**rs689465**			**AA**	**AG**	**GG**	
	Cases	290	202 (0.69)	83 (0.28)	5 (0.03)	0.927
	Controls	244	172 (0.70)	67 (0.27)	5 (0.03)	
						
**rs689466**			**AA**	**AG**	**GG**	
	Cases	289	224 (0.77)	62 (0.21)	3 (0.02)	0.968
	Controls	245	190 (0.77)	51 (0.21)	3 (0.02)	
						
**rs20417**			**GG**	**GC**	**CC**	
	Cases	308	157 (0.51)	127 (0.41)	24 (0.08)	0.731
	Controls	264	129 (0.49)	117 (0.44)	18 (0.07)	
						
**rs5275**			**TT**	**TC**	**CC**	
	Cases	294	125 (0.42)	149 (0.51)	20 (0.07)	**0.046**
	Controls	244	120 (0.49)	99 (0.41)	25 (0.10)	

N: Number of examined samples (with available PCR amplification); * Differences in sample sizes for cases and controls are due to available data in each category. Freq: Frequency; P χ2: P from Chi-square test (Pearson p-value).

The distribution of *PTGS2 *haplotypes among cases and controls was also examined (Table 6). All genotype information was included in the analysis, and the haplotype inference could be obtained for 302 cases and 264 controls. No significant difference in the haplotypic distribution was observed between patients and controls (P = 0.99, Fisher exact method). The three less frequent haplotypes appeared to be differently expressed between cases and controls. However, it was not possible to calculate the OR between cases and controls due to the absence of these haplotypes in one of the groups. An adequate evaluation of their impact on cancer risk would require a much larger sample. To further evaluate the impact of the rs5275 SNP on the risk of developing breast cancer, the haplotypes were separated into groups according to the presence of the rs5275 *T *or *C *allele. No risk association was found for rs5275 *C-*containing haplotypes when considered as a combined group (OR = 1.09, 95% CI = 0.83-1.43; P = 0.5). Taken together, the results appear to indicate no association between *PTGS2 *SNPs (in their most frequent haplotypes) and the risk of breast cancer.

**Table 6:** Haplotype distribution in patients and controls and association with breast cancer risk

		Cases		Controls				
	**Haplotype**	**N**	**F**	**N**	**F**	**OR**	**95%CI**	**P χ2**

	**AAGT**	**284**	**0.47**	**248**	**0.47**	**1**		
								
1	**AAGC**	72	0.12	63	0.12	0.99	0.68-1.45	0.92
	**GACC**	60	0.1	48	0.09	1.09	0.72-1.62	0.68
	**AACC**	54	0.09	42	0.08	1.12	0.72-1.73	0.65
	**GAGC**	6	0.01	0	0			0.03*
	**Total**	**192**	**0.32**	**153**	**0.29**	**1.09**	**0.83-1.43**	**0.5**
								
2	**AGGT**	66	0.11	53	0.1	1.08	0.72-1.62	0.68
	**AACT**	30	0.05	26	0.05	1.00	0.58-1.75	1
	**GACT**	24	0.04	26	0.05	0.80	0.45-1.44	0.46
	**AGCT**	0	0	5	0.01			0.02*
	**GAGT**	0	0	5	0.01			0.02*
	**Others**	8	0.01	12	0.02	0.58	0.23-1.44	0.23
	**Total**	**128**	**0.21**	**127**	**0.24**	**0.88**	**0.65-1.18**	**0.4**
								

	**Total**	**604**	**1**	**528**	**1**			

The haplotype combining the predominant alleles was used as a reference. Group 1 was formed by any haplotype containing the *rs5275 C *allele and group 2 included all the other haplotypes. The haplotypes with less than 1% frequency (Others) are not listed. The impact on breast cancer risk was calculated for the two groups, considering the Odds Ratio (OR) and the 95% Confidence Interval (95%CI) P: Pearson P-value; N: Number of haplotypes. F: Frequency of haplotypes. * Fisher Exact Probability Test (two-tailed). OR was not calculated because of N = 0.

## Discussion

In the past five years, several studies have aimed to evaluate the impact of *PTGS2 *SNPs on the risk of developing different types of cancer [10,15,16,21-46]. However, most of these studies evaluated only one or a few SNPs at a time, sometimes with no clear selection criteria. Zhang et al. [10] were the first to perform a screening strategy to identify the most frequent *PTGS2 *SNPs. This approach was also preferred in our case, due to the heterogeneity of the Brazilian population and to the consequent hazards of using frequency data obtained elsewhere.

In our screening strategy, we evaluated 1.5 kb of the PR and 1.2 kb of the 3'-UTR, which encompass the most important regulatory sites of *PTGS2 *expression [9,10,13]. The focus on the regulatory regions of the gene is justified by the fact that *PTGS2 *mRNA is very unstable [13], and an increase in its stability promotes COX-2 expression in colon cancer cells [13]. In addition, *in vitro *studies indicate possible functional effects of the SNPs rs689466 and rs20417 on *PTGS2 *promoter activity (evaluated by a luciferase gene reporter system) [9,10] and on COX-2 activity (evaluated by PGE2 production in human monocytes) [47]. Although screening the entire *PTGS2 *gene would be theoretically preferable because variants in the coding and non-coding regions could tag other functional SNPs, previous reports involving these variants suggest no significant effect on COX-2 activity [48] or on cancer risk [16,21,25,28-30,32,33,35-43,45,46]. The only exception is a recent publication by Zhao *et al. *[49], which suggests an increased risk of esophageal squamous cancer associated to SNP rs20432 that is located in intron 5. This positive association, however, is not confirmed in other types of cancer [16,21,25,29,33,36,45].

In our screening analysis, we found nine SNPs, five of which showed very low MAFs (approximately 0.01): rs20415, rs20419, rs5270, rs20424 and rs4648298. These MAFs are in accordance with previous reports involving different populations [15,16,50]. The SNPs rs689465, rs689466, rs20417 and rs5275 were selected for the case-control study and appear to be the most frequent SNPs in other Western populations [16,21,23,25,51-58].

The present work is the first study on the frequency of *PTGS2 *SNPs among Brazilians, who are one of the world's most heterogeneous populations as a result of extensive interethnic crosses over the last 500 years between autochthonous Amerindians, European colonizers and Africans [59-61]. Studies based on population-specific alleles, blood groups and electrophoresis of protein markers have outlined the hazards of equating color or race with geographic ancestry in Brazilians [18,59-63]. Thus, the stratification of our population into three groups based on self-reported skin color (white, intermediate and black) was not intended for ethnic classification but to evaluate potential differences in the frequency of *PTGS2 *SNPs due to heterogeneous racial admixture. Because no significant difference in the genotype distribution was detected for the four SNPs among the color groups, either in the general population or among patients (data not shown), no population control or stratification based on continental-specific alleles was necessary in the case-control study.

In the present study, there was no association between rs689465, rs689466 or rs20417 and the occurrence of breast cancer. The results for rs689466 and rs20417 are in accordance with previously published data [23,25,39]. This is the first report on rs689465 and the risk of breast cancer.

Our results show an increase in the frequency of rs5275 *TC *heterozygotes among patients compared to controls, with an apparent increased risk of breast cancer development after adjustment for age differences. The borderline significance of this association, however, limits its confidence. The number of subjects in our case-control study was initially calculated considering the allele frequencies in the general population and a possible 2-fold increase in the rs5275 MAF among patients, with a significance level of 5% and an error level of 20%. Although the actual sample size was larger than first estimated to ensure statistical power, it was still small for the evaluation of inheritance models or for the study of the less frequent haplotypes.

A review of the literature concerning the impact of rs5275 on the risk of breast cancer shows conflicting results. Langsenlehner *et al. *[24] found that carriers of the rs5275 *C *allele in the Austrian population were more frequent among breast cancer patients (34.8%) than among age-matched controls (29.9%; P = 0.018), with an increased risk of breast cancer in rs5275*CC *homozygotes (OR = 2.1; 95% CI = 1.3-3.3; P = 0.002). These results, however, were not corroborated by other authors. Vogel *et al. *[34] found no association between rs5275 genotype and breast cancer susceptibility, which was confirmed in three independent large studies [25,28,39]. Cox *et al. *[25], combining data from three separate studies in the American population (N = 5144), indicated that women homozygous for the rs5275 *C *allele have a 20% lower risk of breast cancer than those homozygous for the *T *allele (OR = 0.80, 95% CI = 0.66-0.97) [25]. This reduced risk was confirmed by Zhu *et al. *[64] in a meta-analysis, which, however, did not include the large number of individuals in the work by Abraham *et al. *[28] and by Dossus *et al. *[39]. Taken together, these studies appear to suggest no strong influence of rs5275 SNP on breast cancer risk.

The present work indicates that variants in the PR and in the 3' UTR of *PTGS2 *do not appear to greatly influence breast cancer risk, as the apparent risk association found for rs5275 SNP was limited to heterozygotes with a low OR value and borderline significance. However, the apparently negative results do not exclude potential low risks (i.e., OR < 1.5), whose detection with high level of statistical significance (P < 0.001) would require large individual studies or meta-analysis (N > 6000). Our data also highlight the existence of various *PTGS2 *haplotypes that have not been thoroughly studied and should be considered for further evaluation of risk association with cancer development and/or progression.

## Conclusion

Our results indicate no strong association between the four most frequent *PTGS2 *SNPs and the risk of breast cancer.

## Competing interests

The authors declare that they have no competing interests.

## Authors' contributions

DNP and RVJ were involved with the rationale, study design, development of genotyping protocols, data analysis and preparation of the manuscript. DNP, JSFV and LMA conducted the recruitment and the all the laboratory procedures. AB and RVJ were responsible for statistical analysis. All authors approved the final manuscript.

## Pre-publication history

The pre-publication history for this paper can be accessed here:

http://www.biomedcentral.com/1471-2407/10/613/prepub

## References

[B1] Needleman P Turk J Jakschik BA Morrison AR Lefkowith JB Arachidonic acid metabolism Annu Rev Biochem 1986 55 69 102 10.1146/annurev.bi.55.070186.000441 3017195

[B2] Smith WL Garavito RM DeWitt DL Prostaglandin endoperoxide synthases (cyclooxygenases)-1 and -2 J Biol Chem 1996 271 33157 160 10.1074/jbc.271.52.33157 8969167

[B3] Zha S Yegnasubramanian ZS Nelson WG Isaacs WB De Marzo AM Cyclooxygenases in cancer: progress and perspective Cancer Letters 2004 205 1 20 10.1016/j.canlet.2004.06.014 15374627

[B4] Ristimaki A Sivula A Lundin J Lundin M Salminen T Haglund C Prognostic significance of elevated cyclooxygenase expression in breast cancer Cancer Res 2002 62 632 635 11830510

[B5] Spizzo G Gastl G Wolf D Gunsilius E Steurer M Fong D Correlation of COX-2 and Ep-CAM overexpression in human invasive breast cancer and its impact on survival British J of Cancer 2003 88 4 574 578 10.1038/sj.bjc.6600741 PMC2377167 12592372

[B6] Pereg D Lishner M Non-steroidal anti-inflammatory drugs for the prevention and treatment of cancer J Intern Med 2005 258 115 23 10.1111/j.1365-2796.2005.01519.x 16018788

[B7] Arun B Goss P The role of COX-2 inhibition in breast cancer treatment and prevention Semin Oncol 2004 31 22 9 10.1053/j.seminoncol.2004.03.042 15179621

[B8] Bundred NJ Barnes NL Potential use of COX-2-aromatase inhibitor combinations in breast cancer British J of Cancer 2005 93 Suppl 1 S10 5 10.1038/sj.bjc.6602690 PMC2361689 16100520

[B9] Papafili A Hill MR Brull DJ McAnulty RJ Marshall RP Humphries SE Common promoter variant in cyclooxygenase-2 repress gene expression Arterioscler Thromb Vasc Biol 2002 22 1631 1636 10.1161/01.ATV.0000030340.80207.C5 12377741

[B10] Zhang X Miao X Tan W Ning B Liu Z Hong Y Identification of functional genetic variants in *Cyclooxygenase-2 *and their association with risk of esophageal cancer Gastroenterology 2005 129 565 576 16083713 10.1016/j.gastro.2005.05.003

[B11] Caput D Beutler B Hartog K Thayer R Brown-Shimer S Cerami A Identification of a common nucleotide sequence in the 3'-unstranlated region of mRNA molecules specifying inflammatory mediators Proc Natl Acad Sci USA 1986 83 1670 4 10.1073/pnas.83.6.1670 2419912 PMC323145

[B12] Di Marco S Hel Z Lachance C Furneaux H Radzioch D Polymorphism in the 3'-unstranlanted region of TNFα mRNA impairs binding of the post-transcriptional regulatory protein HuR to TNFα mRNA Nucleic Acid Res 2001 29 863 71 10.1093/nar/29.4.863 11160917 PMC29616

[B13] Dixon DA Kaplan CD McIntyre TM Zimmerman GA Prescott SM Post-transcriptional Control of Cyclooxygenase-2 Gene Expression The Journal of Biological Chemistry 2000 275 16 11750 11757 10.1074/jbc.275.16.11750 10766797

[B14] Nabors LB Gillespie GY Harkins L King PH HuR, a stability factor, is expressed in malignant brain tumors and binds to adenine- and uridine-rich elements within the 3'-unstranlated regions of cytokine and angiogenic factor mRNAs Cancer Res 2001 61 2154 61 11280780

[B15] Panguluri RCK Long LO Chen W Wang S Coulibaly A Ukoli F COX-2 gene promoter haplotypes and prostate cancer risk Carcinogenesis 2004 25 961 6 10.1093/carcin/bgh100 14754878

[B16] Cox DG Pontes C Guino E Navarro M Osorio A Canzian F Polymorphisms in prostaglandin synthase 2/cyclooxygenase 2 (*PTGS2*/COX2) and risk of colorectal cancer British J of Cancer 2004 91 339 43 10.1038/sj.bjc.6601906 PMC2409800 15173859

[B17] CENSO 2000 http://www.ibge.gov.br/home/estatistica/populacao/censo2000/Accessed Nov 28, 2007

[B18] Parra FC Amado RC Lambertucci JR Rocha J Antunes CM Pena SD Color and genomic ancestry in Brazilians Proc Natl Acad Sci USA 2003 100 177 82 10.1073/pnas.0126614100 12509516 PMC140919

[B19] Raymond M Rousset F GENEPOP (version 1.2): population genetics software for exact tests and ecumenicism J Heredity 1995 86 248 249

[B20] Barrett JC Fry B Maller J Daly MJ Haploview: analysis and visualization of LD and haplotype maps Bioinformatics 2005 [PubMed ID: 15297300] 15297300 10.1093/bioinformatics/bth457

[B21] Campa D Zienolddiny S Maggini V Skaug V Haugen A Canzian F Association of a common polymorphism in the cyclooxygenase 2 gene with risk of non-small cell lung cancer Carcinogenesis 2004 25 229 35 10.1093/carcin/bgh008 14604894

[B22] Sorensen M Autrup H Tjonneland A Overvad K Raaschou-Nielsen O A genetic polymorphism in prostaglandin synthase 2 (*8473*, T/C) and the risk of lung cancer Cancer Letters 2005 226 49 54 10.1016/j.canlet.2005.03.037 15882925

[B23] Shen J Gammon MD Terry MB Teitelbaum SL Neugut AI Santella RM Genetic polymorphisms in the cyclooxygenase-2 gene, use of nonsteroidal anti-inflammatory drugs, and breast cancer risk Breast Cancer Research 2006 6 8 R71 10.1186/bcr1629 PMC1797023 17181859

[B24] Langsenlehner U Yazdani-Biuki B Eder T Renner W Wascher TC Weber B The Cyclooxygenase-2 (*PTGS2*) *8473*T > C Polymorphismis Associated with Breast Cancer Risk Clin Cancer Res 2006 12 4 1392 94 10.1158/1078-0432.CCR-05-2055 16489098

[B25] Cox DG Buring J Hankinson SE Hunter DJ A polymorphism in the 3' untranslated region of the gene encoding prostaglandin endoperoxide synthase 2 is not associated with an increase in breast cancer risk: a nested case-control study Breast Cancer Research 2007 9 1 R3 10.1186/bcr1635 17214885 PMC1851394

[B26] Fernandez P de Beer PM van der Merwe L Heyns CF COX-2 promoter polymorphisms and the association with prostate cancer risk in South African men Carcinogenesis 2008 29 2347 2350 10.1093/carcin/bgn245 18974063

[B27] Upadhyaya R Jain M Kumarb S Ghoshalc UC Mittala B Functional polymorphisms of cyclooxygenase-2 (COX-2) gene and risk for esophageal squmaous cell carcinoma Mutation Research 2009 663 52 59 19428370 10.1016/j.mrfmmm.2009.01.007

[B28] Abraham JE Harrington P Driver KE Tyrer J Easton DF Dunning AM Common polymorphisms in the prostaglandin pathway genes and their association with breast cancer susceptibility and survival Clin Cancer Research 2009 15 6 2181 91 10.1158/1078-0432.CCR-08-0716 19276290

[B29] Danforth KN Hayes RB Rodriguez C Yu K Sakoda LC Huang W-Y Polymorphic variants in *PTGS2 *and prostate cancer risk: results from two large nested case-control studies Carcinogenesis 2008 3 29 568 572 10.1093/carcin/bgm253 17999989

[B30] Li F Ren GS Li HY Wang XY Chen L Li J A novel single nucleotide polymorphism of the cyclooxygenase-2 gene associated with breast cancer Clin Oncol (R Coll Radiol) 2009 4 302 5 10.1016/j.clon.2008.12.005 19138835

[B31] Lin YC Huang HI Wang LH Tsai CC Lung O Dai CY Polymorphisms of COX-2 -765G > C and p53 codon 72 and risks of oral squamous cell carcinoma in a Taiwan population Oral Oncol 2008 44 8 798 804 10.1016/j.oraloncology.2007.10.006 18234542

[B32] Cheng I Liu X Plummer SJ Krumroy LM Casey G Witte JS COX2 genetic variation, NSAIDs, and advanced prostate cancer risk Br J Cancer 2007 97 4 557 61 10.1038/sj.bjc.6603874 17609663 PMC2360347

[B33] Shahedi K Lindström S Zheng SL Wiklund F Adolfsson J Sun J Genetic variation in the COX-2 gene and the association with prostate cancer risk Int J Cancer 2006 119 3 668 72 10.1002/ijc.21864 16506214

[B34] Vogel U Christensen J Nexø BA Wallin H Friis S Tjønneland A Peroxisome proliferator-activated [corrected] receptor-gamma2 [corrected] Pro12Ala, interaction with alcohol intake and NSAID use, in relation to risk of breast cancer in a prospective study of Danes Carcinogenesis 2007 28 9 2062 10.1093/carcin/bgm181 16959787

[B35] Lee TS Jeon YT Kim JW Park NH Kang SB Lee HP Song YS Lack of association of the cyclooxygenase-2 and inducible nitric oxide synthase gene polymorphism with risk of cervical cancer in Korean population Ann N Y Acad Sci 2007 1095 134 42 10.1196/annals.1397.017 17404026

[B36] Gunter MJ Canzian F Landi S Chanock SJ Sinha R Rothman N Inflammation-related gene polymorphisms and colorectal adenoma Cancer Epidemiol Biomarkers Prev 2006 15 6 1126 31 10.1158/1055-9965.EPI-06-0042 16775170

[B37] Siezen CL Bueno-de-Mesquita HB Peeters PH Kram NR van Doeselaar M van Kranen HJ Polymorphisms in the genes involved in the arachidonic acid-pathway, fish consumption and the risk of colorectal cancer Int J Cancer 2006 119 2 297 303 10.1002/ijc.21858 16482563

[B38] Thompson CL Plummer SJ Merkulova A Cheng I Tucker TC Casey G Li L No association between cyclooxygenase-2 and uridine diphosphate glucuronosyltransferase 1A6 genetic polymorphisms and colon cancer risk World J Gastroenterol 2009 15 18 2240 4 10.3748/wjg.15.2240 19437564 PMC2682239

[B39] Dossus L Kaaks R Canzian F Albanes D Berndt SI Boeing H PTGS2 and IL6 Genetic Variation and Risk of Breast and Prostate Cancer: results from the Breast and Prostate Cancer Cohort Consortium (BPC3) Carcinogenesis 2009 31 3 455 61 10.1093/carcin/bgp307 19965896 PMC2832545

[B40] Chang ET Birmann BM Kasperzyk JL Conti DV Kraft P Ambinder RF Polymorphic variation in NFKB1 and other aspirin-related genes and risk of Hodgkin lymphoma Cancer Epidemiol Biomarkers Prev 2009 18 3 976 86 10.1158/1055-9965.EPI-08-1130 19223558 PMC2720066

[B41] Hou L Grillo P Zhu ZZ Lissowska J Yeager M Zatonski W COX1 and COX2 polymorphisms and gastric cancer risk in a Polish population Anticancer Res 2007 27 6C 4243 7 18214026

[B42] Moorman PG Sesay J Nwosu V Kane JG de Cotret AR Worley K Millikan R Cyclooxygenase 2 polymorphism (Val511Ala), nonsteroidal anti-inflammatory drug use and breast cancer in African American women Cancer Epidemiol Biomarkers Prev 2005 14 12 3013 4 10.1158/1055-9965.EPI-05-0291 16365029

[B43] Liu F Pan K Zhang X Zhang Y Zhang L Ma J Genetic variants in cyclooxygenase-2: Expression and risk of gastric cancer and its precursors in a Chinese population Gastroenterology 2006 130 7 1975 84 10.1053/j.gastro.2006.03.021 16762620

[B44] Tan W Wu J Zhang X Guo Y Liu J Sun T Associations of functional polymorphisms in cyclooxygenase-2 and platelet 12-lipoxygenase with risk of occurrence and advanced disease status of colorectal cancer Carcinogenesis 2007 28 6 1197 201 10.1093/carcin/bgl242 17151091

[B45] Ali IU Luke BT Dean M Greenwald P Allellic variants in regulatory regions of cyclooxygenase-2: association with advanced colorectal adenoma Br J Cancer 2005 93 8 953 9 10.1038/sj.bjc.6602806 16205694 PMC1369968

[B46] Sakoda LC Gao YT Chen BE Chen J Rosenberg PS Rashid A Prostaglandin-endoperoxide synthase 2 (PTGS2) gene polymorphisms and risk of biliary tract cancer and gallstones: a population-based study in Shanghai, China Carcinogenesis 2006 27 6 1251 6 10.1093/carcin/bgi314 16361272

[B47] Szczeklik W Sanak M Szczeklik A Functional effects and gender association of COX-2 gene polymorphism G-765C in bronchial Asthma J Allergy Clin Immunol 2004 114 2 248 253 10.1016/j.jaci.2004.05.030 15316498

[B48] Fritsche E Baek SJ King LM Zeldin DC Eling TE Bell DA Functional Characterization of Cyclooxygenase-2 Polymorphisms The Journal of Pharmacology and Experimental Therapeutics 2001 299 2 468 76 11602656

[B49] Zhao D Xu D Zhang X Wang L Tan W Guo Y Interaction of cyclooxygenase-2 variants and smoking in pancreatic cancer: a possible role of nucleophosmin Gastroenterology 2009 136 5 1659 68 10.1053/j.gastro.2009.01.071 19422084

[B50] SeattleSNP Seattle, WA: NHLBI Program for Genomic Applications http://pga.gs.washington.edu (accessed every month between Jan - Oct/2008)

[B51] Gallicchio L Mcsorley MA Newschaffer CJ Huang HY Hoffman SC Helzlsouer KJ Nonsteroidal antiinflammatory drugs, cyclooxygenase polymorphisms, and the risk of developing breast carcinoma among women with benign breast disease Cancer 2006 106 7 10.1002/cncr.21763 16502408

[B52] Ulrich CM Whitton J Yu JH Sibert J Sparks R Potter JD *PTGS2 *(COX-2) *-765*G > C Promoter Variant Reduces Risk of Colorectal Adenoma among Nonusers of Nonsteroidal Anti-inflammatory Drugs Cancer Epidemiol biomarkers Prev 2005 14 3 10.1158/1055-9965.EPI-04-0510 15767339

[B53] Vogel U Christensen J Walin H Friis S Nexø BA Tjønneland A Polymorphisms in *COX-2*, NSAID use and risk of basal cell carcinoma in a prospective study of Danes Mutation Research 2007 617 138 146 17307204 10.1016/j.mrfmmm.2007.01.005

[B54] Lira MG Mazzola S Tessari SG Malerba G Ortombina M Nald L Association of functional gene variants in the regulatory regions of COX-2 gene (*PTGS2*) with nonmelanoma skin cancer after organ transplantation Br J Dermatol 2007 157 1 49 57 10.1111/j.1365-2133.2007.07921.x 17578436

[B55] Hakansson A Bergman O Chrapkowska C Westberg L Belin AC Sydow O Cyclooxygenase-2 polymorphisms in parkinson's disease American Journal of Medical Genetics Part B (Neuropsychiatric Genetics) 2007 144B 367 369 10.1002/ajmg.b.30449 17171651

[B56] Hegener HH Diehl KA Kurth T Gaziano JM Ridker PM Zee RYL Polymorphisms of prostaglandin-endoperoxide synthase 2 gene, and prostaglandin-E receptor 2 gene, C-reactive protein concentrations and risk of atherothrombosis: a nested case-control approach Journal of Thrombosis and Haemostasis 2006 4 1718 1722 10.1111/j.1538-7836.2006.02054.x 16879213

[B57] Kohsaka S Volcik KA Folsom AR Wu KK Ballantyne CM Willerson JT Increased risk of incident stroke associated with the cyclooxygenase 2 (COX-2) G-765C polymorphism in African-Americans: the atherosclerosis risk in communities study shun Atherosclerosis 2008 196 926 930 10.1016/j.atherosclerosis.2007.02.010 17350020

[B58] Abdullah L Ait-Ghezala G Crawford F Crowell TA Barker WW Duara R The cyclooxygenase 2 *-765C *promoter allele is a protective factor for Alzheimer's disease Neuroscience Letters 2005 16309832 10.1016/j.neulet.2005.10.090

[B59] Salzano FM Freire-Maia N Problems in human biology -- a study of Brazilian populations 1970 Detroit: Wayne State University Press 54 8

[B60] Dornelles CL Callegari-Jacques SM Robinson WM Weimer TA Franco MHLP Hickmann AC Genetics, surnames, grandparents' nationalities and ethnic admixture in Southern Brazil: do the patterns of variation coincide? Genet Mol Biol 1999 22 151 61 10.1590/S1415-47571999000200003

[B61] Alves-Silva J Santos MS Guimarães PE Ferreira AC Bandelt HJ Pena SD The ancestry of Brazilian mtDNA lineages Am J Hum Genet 2000 67 444 61 10.1086/303004 10873790 PMC1287189

[B62] Sans M Admixture studies in Latin America: from the 20th to the 21st century Hum Biol 2000 72 155 77 10721616

[B63] Krieger H Morton NE Mi MP Azevedo E Freire-Maia A Yasuda N Racial admixture in north-eastern Brazil Ann Hum Genet 1965 29 113 25 10.1111/j.1469-1809.1965.tb00507.x 5863835

[B64] Zhu W Wei BB Shan X Liu P-765G > C and *8473*T > C polymorphisms of COX-2 and cancer risk: a meta-analysis based on 33 case-control studies Mol Biol Rep 2009 in press 19669667 10.1007/s11033-009-9685-1

